# The effect of COVID-19 on public confidence in the World Health Organization: a natural experiment among 40 countries

**DOI:** 10.1186/s12992-022-00872-y

**Published:** 2022-08-20

**Authors:** Chao Guo, Xiyuan Hu, Dianqi Yuan, Yuyu Zeng, Peisen Yang

**Affiliations:** 1 Institute of Population Research, Peking University, 100871 Beijing, China; 2 APEC Health Science Academy (HeSAY), Peking University, Beijing, 100871 China

**Keywords:** COVID-19, Pandemic, Public confidence, Trust, World Health Organization

## Abstract

**Background:**

At a time when a highly contagious pandemic and global political and economic turmoil are intertwined, worldwide cooperation under the leadership of an international organization has become increasingly important. This study aimed to estimate the effect of COVID-19 on public confidence in the World Health Organization (WHO), which will serve as a reference for other international organizations regarding the maintenance of their credibility in crisis management and ability to play a greater role in global health governance.

**Methods:**

We obtained individual data from the World Values Survey (WVS). A total of 44,775 participants aged 16 and older from 40 countries in six WHO regions were included in this study. The COVID-19 pandemic was used as a natural experiment. We obtained difference-in-differences (DID) estimates of the pandemic’s effects by exploiting temporal variation in the timing of COVID-19 exposure across participants interviewed from 2017 to 2020 together with the geographical variation in COVID-19 severity at the country level. Public confidence in the WHO was self-reported by the respondents.

**Results:**

Among the participants, 28,087 (62.73%) reported having confidence in the WHO. The DID estimates showed that the COVID-19 pandemic could significantly decrease the likelihood of people reporting confidence in the WHO after controlling for multiple covariates (adjusted OR 0.54, 95% CI: 0.49–0.61), especially during the global outbreak (0.35, 0.24–0.50). The effect was found in both younger individuals (0.58, 0.51–0.66) and older adults (0.49, 0.38–0.63) and in both males (0.47, 0.40–0.55) and females (0.62, 0.53–0.72), with a vulnerability in males (adjusted P for interaction = 0.008).

**Conclusion:**

Our findings are relevant regarding the impact of COVID-19 on people’s beliefs about social institutions of global standing, highlighting the need for the WHO and other international organizations to shoulder the responsibility of global development for the establishment and maintenance of public credibility in the face of emergencies, as well as the prevention of confidence crises.

**Supplementary Information:**

The online version contains supplementary material available at 10.1186/s12992-022-00872-y.

## Background

The outbreak of COVID-19 has infected 274 million people and taken 5.35 million lives as of December 20, 2021 [1]. The pandemic continues to challenge people’s physical and mental health with highly transmissible SARS-CoV-2 variants [2], resulting in increasing health disparities [3]. It has also had a great impact on the economy, increasing both poverty and unemployment [4]. The pandemic is making life harder for the vulnerable. The well-being of women especially deserves more attention given systematic barriers (e.g., sexism) in access to resources and their position as a majority of front-line healthcare workers [5]. The duration of this pandemic is unprecedented in modern times, leading to social unrest throughout the world.

The World Health Organization (WHO), founded in 1948, was the first United Nations agency devoted to global health affairs. The Constitution of the World Health Organization prescribes the rights and obligations of WHO to assist all peoples in attaining the highest possible level of health [6]. A central and historic duty of the WHO has been the management of the global regime for the control of international public health crises [7]. The International Health Regulations (IHR) approved in 2005 stipulate the responsibilities and obligations of WHO and member states in disease prevention, including defence and control of the international spread of disease and the provision of public health response measures [8]. Since then, as “the only source of legally binding international regulations for pandemic response” [9], the WHO has played an increasingly important role in preventing the spread of disease between countries as evidenced by its response to the 2009 pandemic influenza A(H1N1) virus, polio in 2014, Zika in 2014, Ebola in 2014 and 2018, and COVID-19 in 2020 [10].

The IHR, together with other instruments, such as the Global Outbreak Alert and Response Network (2000), the Pandemic Influenza Preparedness Framework (2011), the Public Health Emergency Operations Centre Network (2012), and the Contingency Fund for Emergencies (2015), also help WHO strengthen national public health systems [9]. In response to the pandemic, the WHO plays a key role in two aspects: sharing the Health Emergencies Programme and building the Health System. The WHO Health Emergencies Programme has made a considerable impact in the world, taking a stronger operational role. The Health Emergencies Programme includes preventing epidemics and pandemics and responding to health emergencies [11]. Throughout the programme, tests, treatments, and vaccines can be researched in a timely manner, essential supplies shipped to countries, and the healthcare workforce can be protected and trained. In 2019, the WHO responded to 55 emergencies in more than 44 countries and territories [10]. Several pandemics in the past have reminded us of the importance of preparedness, a strong health system that is resilient to shock, and the need to ensure systems that can maintain essential health services without financial hardship, especially during times of crisis. The WHO reiterated its commitment to supporting countries as they build universal health coverage. By 2019, 91 countries had improved patient safety, and 42 countries had implemented national healthcare workforce accounts [10].

During COVID-19, the WHO provides frontier support in leadership, policy dialogue and strategic support, as well as technical assistance and service delivery [12]. After the Wuhan Municipal Health Commission reported the cluster of atypical pneumonia cases, the WHO set up the Incident Management Support Team on January 1, 2020, to deal with the outbreak. At the IHR Emergency Committee meeting held on January 30, 2020, the WHO declared the novel coronavirus outbreak a Public Health Emergency of International Concern (PHEIC) and helped to establish national and international emergency coordination mechanisms [13]. The WHO has taken measures to respond to COVID-19 under a tight budget, such as convening an expert panel to develop interim best practice guidance for vaccine efficacy evaluations [14]. By December 31, 2020, 91% of countries had a COVID-19 preparedness and response plan, and 97% had a functional COVID-19 coordination mechanism [15]. The WHO also published the Strategic Preparedness and Response Plan aimed at controlling the spread of the virus and provided technical assistance, including deploying Emergency Medical Teams, establishing a global surveillance system, and working with partner laboratories [15].

Although Article 66 of the WHO Constitution requires legal capacity in the territory of each member [16] and the IHR states that “If a PHEIC is declared, WHO develops and recommends the critical health measures for implementation by the Member States during such an emergency” [7], these “soft laws” fall short of binding responsibilities [9], and the review committee has noted that “the IHR has no teeth” [17]. Some countries with weaker health systems are unable to follow the instructions of the WHO well [18]. The WHO has also received much criticism, including the irrationality of the WHO’s workplace health and safety guidelines on COVID-19 [19] and the inability to address the needs of older people [20]. The broad criticism, is somewhat unfair [10] since the failure to control the COVID-19 outbreak in the early stages was led by the inefficient early alarm and inadequate compliance of states with obligations under the IHR together [21]. A potential crisis of trust in the WHO is especially harmful given that the pandemic poses a threat to vulnerable people and regions. Nevertheless, it can be clearly realized that the WHO began to refashion itself as the coordinator, strategic planner, and leader of global health initiatives despite facing budget shortfalls and diminished status, especially given the growing influence of new and powerful players [22].

Only when people trust the WHO will they listen to its advice on pandemic prevention and control and promote global cooperation. It is worth noting that trust in social institutions is associated with the adoption of preventive behaviours during the pandemic [23–26], and health awareness and behaviours are undoubtedly necessary protective measures. A previous study among Americans found that trust in the competence of the WHO could play an important role in preventive health behaviours in addition to trust in the U.S. Centers for Disease Control and Prevention (CDC) [27]. Trust in the WHO has been under attack in recent years. In addition to the aforementioned, the level of public confidence in the WHO is influenced to some extent by the following.

The global political situation influenced people’s trust in the WHO because the WHO is funded by a combination of members' fees based on wealth and population and voluntary contributions. The news that then-President Donald Trump moved to withdraw the United States from the WHO on July 7, 2020 would call into question the WHO's financial viability and the future of its many programs promoting healthcare and tackling disease [28]. Second, the lack of a strong accountability mechanism, leading to the inadequate compliance of states with obligations under the IHR, probably caused people to lose confidence in the WHO [21]. For example, India refused to cooperate with the WHO to deal with H5N1 influenza in 2007 [29]. This is a clear violation of the IHR's obligation to minimally intervene and the member's obligation to cooperate, but there are no punitive measures under the IHR. Last, it is crucial to increase or at least maintain the quality and speed of health services or crisis management to maintain confidence in the WHO. A study in Korea demonstrated that the improvement in trust in central and local government is associated with proactive responses to the pandemic crisis, while the deteriorating trust in religious organizations is a consequence of their late approach to the crisis [30]. Given the important role of the WHO in global health governance, although many efforts have been made, a public health emergency that has not been effectively prevented and controlled, evidenced by increasing morbidity and mortality, is likely to lead to a decline in people's confidence in the WHO.

A longitudinal investigation researched the evolution of public trust in institutions during and after the 2009 pandemic in Switzerland and found that trust in almost all institutions decreased between the beginning of the outbreak and a year later. The magnitude of the decrease was particularly high for the WHO and the pharmaceutical industry benefiting from a relatively high level of initial trust [31]. Although some scholars who analysed people’s trust in science during the pandemic and found the overall level of trust in science remained unchanged after the first several months of COVID-19 [32], trustworthiness in COVID-19 information sources, such as the mainstream media, state health departments, the CDC, the White House, and a well-known university declined significantly in the United States [33]. However, considering the importance of the WHO during this pandemic, the effect of COVID-19 on public confidence in the WHO has yet to be well explored.

In this study, we used COVID-19 as a natural experiment to examine whether this pandemic has caused a crisis of confidence in the WHO. To be more specific, we adopted a difference-in-differences (DID) method that composited variations of trust in both time and space during COVID-19 to estimate the influence of COVID-19 on public confidence in the WHO. It can provide implications for the far-ranging effect of the public health emergency on people’s beliefs, including trust in leading international organizations. It can also shed light on the high priority that the WHO and other international organizations should place on the global development, the establishment and maintenance of public credibility in the face of emergencies and the prevention of crises in confidence.

## Methods

### Study participants

We obtained individual data from the World Values Survey (WVS) wave 7 conducted from 2017 to 2020 [34]. The WVS is an international research program that aims to collect a wide range of information on the social, political, economic, religious, and cultural values of people in the world. As the largest non-commercial, cross-national, time series investigation of human beliefs and values, the survey from 2017 to 2020 consists of nationally representative surveys conducted in 77 countries and societies on all inhabited continents around the globe using a common questionnaire.

**Fig. 1:**
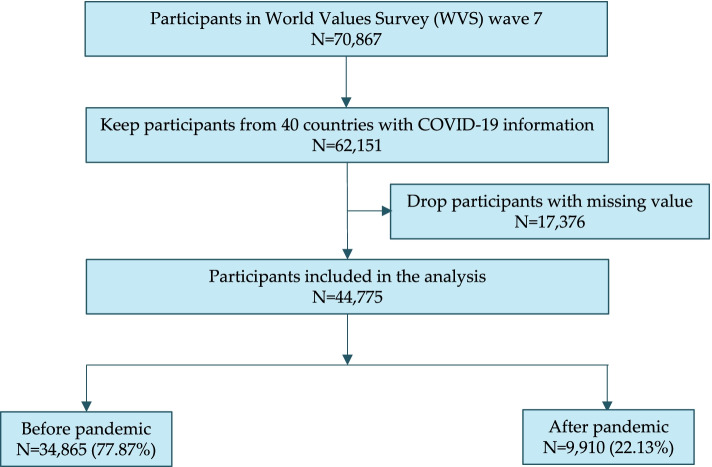
Flowchart of samples

Data on the daily COVID-19 cases and deaths in each country, area, or territory were obtained from the WHO COVID-19 Detailed Surveillance Data [1]. We combined the COVID-19 data and WVS and kept participants whose information was included in both sources. Figure 1 illustrates the derivation of our analytical sample. In the WVS, 70,867 participants were interviewed. Among them, 62,151 participants from countries with COVID-19 information were retained for analysis. Then, we eliminated 15,636 participants who had missing data on the outcome measure or any covariate, and 44,775 participants aged 16 years and above from 40 countries in 6 WHO regions were included in the final sample (Additional file 1: Appendix Table S1).

### Measure

#### Exposure

The global COVID-19 pandemic was used as a natural experiment. The exposure in our study is any possible exposure to the COVID-19 pandemic (not exposure to infection) since the beginning of 2020. We measured it by temporal variation in the timing of the pandemic across interview windows, along with geographical variation in COVID-19 severity at the country level.

Participants who were interviewed from January to the end of 2020 experienced COVID-19 as a PHEIC and were defined as the exposed (after-pandemic) group. Those being interviewed before the outbreak of COVID-19 during the period 2017–2019 served as the reference (before-pandemic) group. Additionally, given that the WHO declared the COVID-19 outbreak a global pandemic on March 11, 2020, we further categorized those interviewed after the pandemic as part of the local epidemic group (interviewed from January to March 2020) and the global pandemic group (interviewed after April 2020).

To measure the severity of COVID-19, we first derived a country-level index of incidence. It was calculated by cumulative cases of COVID-19 from the publicly available WHO COVID-19 Detailed Surveillance Data and the national population size from the World Population Prospects 2019 [35] and was standardized to avoid the influence of dimensions.

Let ${N}_{total}^{j}$ denote the total population of the *j*th country in 2020, and let ${N}_{cumulative cases}^{j}$ denote the cumulative number of COVID-19 cases in the *j*th country. In the WHO database, all cases in our analysis are reported during 2020. The incidence of COVID-19 in 2020 for the *j*th country is calculated as a ratio of ${N}_{cumulative cases}^{j}$ and ${N}_{total}^{j}$. Then, let $\mu$ and $\sigma$ denote the mean and standard deviation of the ratio above, respectively, and ${SI}_{j}$, the standardized incidence of COVID-19 can be obtained from:

$${SI}_{j}=(\frac{{N}_{cumulative\,cases}^{j}}{{N}_{total}^{j}}\times 100\%-\mu )/\sigma$$

where a larger value indicates a more severe COVID-19 pandemic. In addition, we also derived an alternative index of standardized mortality (*SM*) to substitute for the *SI*. It was calculated with the cumulative deaths instead of the cumulative cases using the following formula.

$${SM}_{j}=(\frac{{N}_{cumulative\,deaths}^{j}}{{N}_{total}^{j}}\times 100\%-\mu )/\sigma$$

Given the biases in COVID-19 mortality overestimation indicated by previous studies [36], we only used *SM* for sensitivity analysis.

### Outcomes of interest

The outcome variable in this study was public confidence in the WHO. We constructed a dichotomous variable “confidence in the WHO” (yes or no) according to the answers of participants to the question “How much confidence you have in the WHO?” in the WVS. Answers of “a great deal of confidence” or “quite a lot of confidence” were defined as “yes”, and answers of “not very much confidence” or “none at all” were defined as “no”.

### Covariates

Control variables included some potential confounding factors that were connected with general trust or trust in organizations, including religious value (religious person, not religious person, or atheist) [37], attitude towards science (negative or positive) [38], interest in politics (yes or no) [39] and daily social media user (yes or no) [40, 41]. We also included demographic and socioeconomic status as covariates in the models. These included exact age, sex (male or female), marital status (married or living together as married, or otherwise), residence (rural or urban), international immigrant (yes or no), education level (lower, middle, or higher), employment status (paid employment or other) and income level (low, medium, or high). Further details for each variable are available in Additional file 2: Appendix Table S2.

### Statistical analyses

We employed a difference-in-differences (DID) method that exploited both temporal and geographical variations in COVID-19 exposure to estimate the effects of COVID-19 on public confidence in the WHO. Let ${y}_{ijk}$ denote an outcome of confidence in the WHO for the *i*th participant interviewed in *j*th period and country with *k*th COVID-19 severity, ${X}_{ijk}$ denote the covariates if any, and let ${\varepsilon }_{ijk}$ be a random error. Logit Regression Models with DID estimator can be obtained as follows:

$${Y}_{ijk}={\alpha }_{0}+{\alpha }_{1}{Period}_{j}+{\alpha }_{2}{Severity}_{k}+\beta {(Period}_{j}\times {Severity}_{k})+\gamma {X}_{ijk}+{\varepsilon }_{ijk}$$

where $\beta$, the coefficient of the interaction between period and COVID-19 severity, is the DID estimate of COVID-19 exposure on public confidence in the WHO. It represents the average COVID-19 effect across countries on public confidence in the WHO corresponding to a one-unit change above or below the average severity of COVID-19.

In each model, we first calculated the crude odds ratio (OR) and 95% confidence interval (CI) without covariates and then adjusted the estimators by controlling for the covariates. *P* values were calculated based on robust standard errors that adjust for the potential correlation of observations clustered within the same countries. A two-sided *P* value of less than 0.05 was identified as statistically significant in this study. STATA 16 (STATA Corp, College Station, TX, USA) was used for data analysis.

## Results

### Sample characteristics

The average age at the time of survey for the 44,775 participants in the analysis was 41.72 ± 15.99 years. Among them, 48.92% were males, 65.03% resided in urban areas, 64.33% were married or lived together as married, and 59.61% had paid employment. Table 1 displays the descriptive statistics of the participants by interview period. The two groups interviewed before and after the pandemic resembled each other in terms of sex composition but had differences in other covariates.

**Table 1:** Characteristics of samples

Characteristics	Total	Interviewed period		*P* value
		Before-pandemic	After- pandemic	
Total sample (N)	44,775	34,865	9,910	
Age, years (mean, SD)	41.72 (15.99)	42.22 (16.07)	39.98 (15.57)	< 0.001
Sex (N, %)				0.569
Male	21,904 (48.92)	17,081 (48.99)	4823 (48.67)	
Female	22,871 (51.08)	17,784 (51.01)	5087 (51.33)	
Marital status (N, %)				< 0.001
Married or living together as married	28,805 (64.33)	22,098 (63.38)	6,707 (67.68)	
Otherwise	15,970 (35.67)	12,767 (36.62)	3,203 (32.32)	
Residence (N, %)				< 0.001
Urban	29,115 (65.03)	24,367 (69.89)	4,748 (47.91)	
Rural	15,660 (34.97)	10,498 (30.11)	5,162 (52.09)	
International Immigrant (N, %)				< 0.001
Yes	2,005 (4.48)	1,758 (5.04)	247 (2.49)	
No	42,770 (95.52)	33,107 (94.96)	9,663 (97.51)	
Highest educational level (N, %)				0.006
Lower	14,771 (32.99)	11,392 (32.67)	3,379 (34.10)	
Middle	15,410 (34.42)	12,120 (34.76)	3,290 (33.20)	
Higher	14,594 (32.59)	11,353 (32.56)	3,241 (32.70)	
Employment status (N, %)				< 0.001
Have paid employment	26,692 (59.61)	20,943 (60.07)	5,749 (58.01)	
Otherwise	18,083 (40.39)	13,922 (39.93)	4,161 (41.99)	
Income level (N, %)				< 0.001
Low	11,741 (26.22)	8,962 (25.70)	2,779 (28.04)	
Medium	28,707 (64.11)	22,580 (64.76)	6,127 (61.83)	
High	4,327 (9.66)	3,323 (9.53)	1,004 (10.13)	
Religious value (N, %)				< 0.001
Religious person	31,461 (70.26)	24,437 (70.09)	7,024 (70.88)	
Not religious person	11,072 (24.73)	8,527 (24.46)	2,545 (25.68)	
Atheist	2,242 (5.01)	1,901 (5.45)	341 (3.44)	
Attitude to science (N, %)				< 0.001
Negative	12,403 (27.70)	9,978 (28.62)	2,425 (24.47)	
Positive	32,372 (72.30)	24,887 (71.38)	7,485 (75.53)	
Interested in politics (N, %)				< 0.001
Yes	20,605 (46.02)	15,656 (44.9)	4,949 (49.94)	
No	24,170 (53.98)	19,209 (55.1)	4,961 (50.06)	
Daily social media user (N, %)				< 0.001
Yes	19,794 (44.21)	15,624 (44.81)	4,170 (42.08)	
No	24,981 (55.79)	19,241 (55.19)	5,740 (57.92)	

### Public confidence in the WHO

**Fig. 2:**
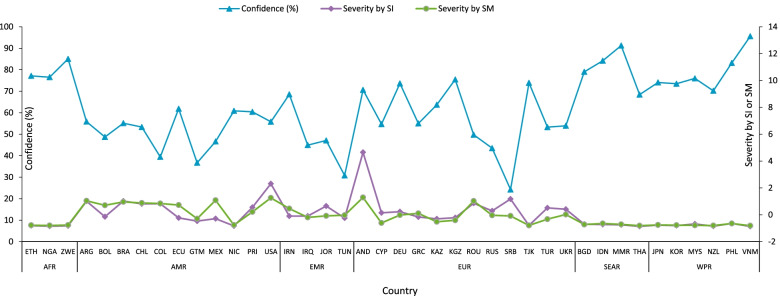
The line graph of confidence in WHO (%) and COVID-19 severity by SI as well as severity by SM. The horizontal label is a shorthand for each country in the WVS according to the standard of ISO 3166–1 alpha 3 code (https://www.nationsonline.org/oneworld/country_code_list.htm) and can also be found in Additional file 1: Appendix Table S1. WHO regions: AFR, African Region; AMR, Region of the Americas; EMR, Eastern Mediterranean Region; EUR, European Region, SEAR, South-East Asia Region; WPR, Western Pacific Region

Among the participants, 28,087 (62.73%) reported having confidence in the WHO, and the proportion among persons interviewed after the pandemic (75.50%) was higher than that among those interviewed before the pandemic (59.10%), without further stratifying each group by a degree of COVID-19 severity in each country. Although a higher proportion of having confidence was observed in the after-pandemic group, it was unclear whether this finding was due to the effect of the pandemic or due to other causes that occurred during this period or other characteristics of the group. Figure 2 shows a negative correlation between public confidence in the WHO and the severity of the COVID-19 pandemic. Specifically, living in areas with higher *SIs* (OR: 0.77, 95% CI: 0.75–0.79) or *SMs* (OR: 0.59, 95% CI: 0.57–0.60) was associated with lower WHO confidence.

### Effect of COVID-19 on public confidence in the WHO

**Fig. 3:**
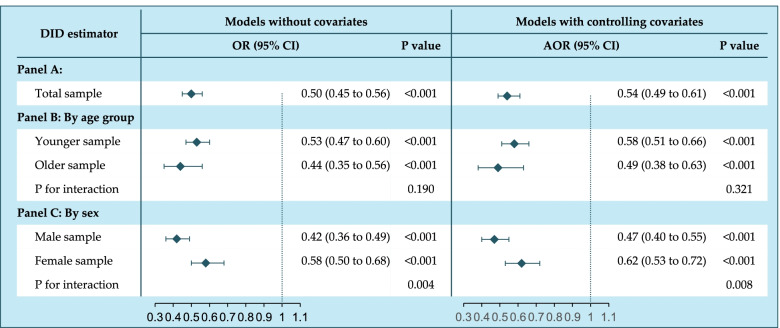
The effect of COVID-19 on public confidence in the WHO. OR, the odds ratio; AOR, the adjusted odds ratio after controlling for covariates including exact age, sex, marital status, residence, international immigrant, education level, employment status, income level, religious value, attitude to science, interested in politics, and daily social media user

The DID estimates shown in Panel A of Fig. 3 further indicated that exposure to the COVID-19 pandemic harmed the WHO in terms of public trust by significantly decreasing the likelihood of people reporting confidence in the organization by half (OR: 0.50, 95% CI: 0.45–0.56). The effect was still significant after controlling for multiple covariates (adjusted OR: 0.54, 95% CI: 0.49–0.61).

### Population heterogeneity by age and sex

The potential heterogeneity of the effect across the population was examined by age group and sex. After stratifying by age group (Panel B of Fig. 3), both younger individuals aged 16–59 years (0.58, 0.51–0.66) and older adults aged 60 years or older (0.49, 0.38–0.63) presented a lower likelihood of confidence in the WHO after exposure to COVID-19, and the difference in effect between the above two subsamples was not statistically significant (adjusted P for interaction = 0.321).

In terms of sex difference (Panel C of Fig. 3), significant effects were found in both the male sample (0.47, 0.40–0.55) and female sample (0.62, 0.53–0.72) in the multiple models, and the likelihood of confidence declined more in males (adjusted P for interaction = 0.008).

### Robustness tests

We performed a series of robustness checks (Additional file 3: Appendix Table S3). First, we used SM instead of SI to measure the severity of COVID-19 and performed the DID analysis again, and we still observed a significantly negative effect of the pandemic on confidence in the WHO after controlling for covariates (0.82, 0.74–0.90). Second, to test whether there was a nonlinear association in the models, we especially controlled for the quantitative predictor, i.e. age, using fractional polynomial models with all other covariates remaining, and a similar effect was found (0.55, 0.49–0.61). Third, we dropped participants from the United States, and the DID analysis also presented similar results (adjusted OR: 0.57, 0.51–0.64). Fourth, we analysed the effect by the stage of development of the pandemic. After controlling for covariates, exposure to the local epidemic and global pandemic both presented lower confidence in the WHO, and the decline in likelihood was especially higher in the global pandemic period (0.35, 0.24–0.50).

## Discussion

The findings presented here indicate that the coronavirus pandemic significantly jeopardized public confidence in the WHO, especially during the pandemic period. A descriptive study on Pan-European trust in WHO preventive measures also found that countries severely affected by COVID-19 reported lower levels of trust [42]. A previous study in the United States showed that Democrats, liberals, and those with a strong cooperative internationalist foreign policy orientation rather than Republicans, conservatives, and nationalists tended to trust the WHO's competence and integrity in dealing with the pandemic [27]. The fast-moving pandemic is one of the important reminders that we are all connected, and the WHO is playing an increasingly important role in promoting consensus and cooperation given that the destiny of humankind is intrinsically shared. It is significant and urgent to engage people around the world to build trust in the WHO. Additionally, trust is earned [43], and the WHO should pay attention to improving its credibility.

Our findings on the negative impact of COVID-19 on WHO credibility may be explained by the following. First, it is plausible that individual attitudes towards the WHO are largely influenced by government attitudes towards the WHO. Science and politics are generally intertwined [43]. Some research has pointed out that evaluations of pandemic responses are becoming increasingly political and that beliefs about scientists' practices and presidents’ opinions are central to the science-politics nexus during pandemics [44]. The Wellcome Global Monitor COVID-19 survey found that trust in scientists squared with trust in national governments, and global trust in science and scientists has increased but with enormous regional differences [45]. The intersection of science and politics gives the government's attitude towards the WHO a higher weight in determining public trust. Government agencies' ignorance and misinterpretation of the WHO's guidance, recommendations, and initiatives, as well as selective adoption or inability to put them into practice, could make people underestimate or doubt the WHO's role in responding to global crises.

It is also important to note that news media coverage of how the WHO responds to the virus can also affect public trust. The pandemic H1N1 of 2009–2010 was hyped in news coverage and potentially affected confidence in pandemic messaging and response activities [46]. In addition, the “infodemic” named by the WHO, which means an overload of information especially false and harmful messages during an outbreak of disease [47], can also sway people's judgement, discredit health authorities and worsen outbreaks. In summary, vaccination and control measures such as stay-at-home/shelter-in-place orders, media trust [48], partisanship [49], and so on played a role in shaping individual responses to the COVID-19 pandemic, which can all affect people’s beliefs about the WHO because much information on the pandemic and advice is thought to be given by this international organization. The comprehensive results of government attitudes and media reports could eventually be reflected in the COVID-19 morbidity and mortality rates, which will affect people's trust in the WHO, one of the organizations entrusted with putting an end to the global pandemic.

In addition, we found that the decline in public confidence in the WHO was more pronounced in males than in females. This may be partially explained by the difference between males and females in compliance behaviours. Previous studies indicated that familiarity with and adherence to the WHO preventive measures were higher among females than males [42], while noncompliance, especially with hygiene-related measures, was more prevalent in males [50]. Furthermore, according to the WHO, the global sex ratio (male/female) of deaths and case fatality rate were 1.39 and 1.40, respectively, by the end of 2020 [1]. This offers further insight into the sources of the vicious cycle between distrust of the WHO, noncompliance with quarantine measures, and high risk of death among males.

### Limitations

This study had several limitations. First, some factors that we did not control may potentially confound our results, such as countries' media environment and the coverage of the WHO during 2020. Second, we explored the general level of trust in the WHO instead of specific dimensions of trust. Focusing on specific content, such as pandemic control strategies, cooperation, and support for countries and regions in trouble, may help us understand people’s beliefs and feelings about the WHO more comprehensively. Third, given that the grouping of exposure periods in our analyses is in years or months, our results reflect an averaging effect and should thus be interpreted with caution. Fourth, we measured the effects of COVID-19 on public trust in the WHO only in the early and most severe phases of the global outbreak that occurred until the end of 2020. It is still unclear what the trend in public confidence is during this ongoing pandemic. Fifth, although counts of cases and deaths are from the WHO, they are based on the integration of official reports from various countries, and some bias may come from different standards. Despite these limitations, to the best of our knowledge, this is the first study to examine the impact of COVID-19 on public trust in the WHO at the global level based on robust data and methods.

## Conclusions

We found that COVID-19 was associated with a decline in people’s trust in the WHO at the onset of the COVID-19 outbreak, which raises the risk that such a global health crisis could undermine public trust in the WHO. Of note, the confidence of men declined more than that of women. Our findings showed the importance and urgency of taking measures to restore public trust in the WHO, to support its capacity as a multilateral institution to coordinate action during a global health crisis. This research also contributes new knowledge to the developing body of work exploring the impact of the pandemic on trust in public institutions more broadly.

## Supplementary Information

**Additional file 1: Appendix Table S1.** Country list in analysis. **Appendix Table S2. **Measurement of covariates. **Appendix Table S3.** The effect of COVID-19 on public confidence in the WHO: robustness tests.

## Data Availability

The datasets generated and/or analyzed during the current study are available in the WVS repository, https://www.worldvaluessurvey.org/WVSDocumentationWV7.jsp.

## Abbreviations

WHO: World Health Organization
IHR: International Health Regulations
PHEIC: Public Health Emergency of International Concern
CDC: Centers for Disease Control and Prevention
DID: Difference-in-differences
WVS: World Values Survey
SI: Standardized incidence
SM: Standardized mortality
OR: Odds ratio
CI: Confidence interval
