# Supplementary material for: The effect of COVID-19 on public confidence in the World Health Organization: a natural experiment among 40 countries
Source: Global Health. 2022 Aug 20;18:77. doi: 10.1186/s12992-022-00872-y (PMC9392065; doi:10.1186/s12992-022-00872-y)
Supplement: Supplementary file 1 — Additional file 1: Appendix Table S1. Country list in analysis. Appendix Table S2. Measurement of covariates. Appendix Table S3. The effect of COVID-19 on public confidence in the WHO: robustness tests. [file 12992_2022_872_MOESM1_ESM.docx]

**Appendix Table S1.** Country list in analysis.

| WHO region | Country name | ISO 3166 1 alpha 3 code |
| --- | --- | --- |
| African Region (AFR) | Ethiopia | ETH |
|  | Nigeria | NGA |
|  | Zimbabwe | ZWE |
| Region of the Americas (AMR) | Argentina | ARG |
|  | Bolivia | BOL |
|  | Brazil | BRA |
|  | Chile | CHL |
|  | Colombia | COL |
|  | Ecuador | ECU |
|  | Guatemala | GTM |
|  | Mexico | MEX |
|  | Nicaragua | NIC |
|  | Puerto Rico | PRI |
|  | United States of America | USA |
| Eastern Mediterranean Region (EMR) | Iran | IRN |
|  | Iraq | IRQ |
|  | Jordan | JOR |
|  | Tunisia | TUN |
| European Region (EUR) | Andorra | AND |
|  | Cyprus | CYP |
|  | Germany | DEU |
|  | Greece | GRC |
|  | Kazakhstan | KAZ |
|  | Kyrgyzstan | KGZ |
|  | Romania | ROU |
|  | Russian Federation | RUS |
|  | Serbia | SRB |
|  | Tajikistan | TJK |
|  | Turkey | TUR |
|  | Ukraine | UKR |
| South-East Asia Region (SEAR) | Bangladesh | BGD |
|  | Indonesia | IDN |
|  | Myanmar | MMR |
|  | Thailand | THA |
| Western Pacific Region (WPR) | Japan | JPN |
|  | Republic of Korea | KOR |
|  | Malaysia | MYS |
|  | New Zealand | NZL |
|  | Philippines | PHL |
|  | Viet Nam | VNM |

Note: the standard of ISO 3166 1 alpha 3 code can be seen at <https://www.nationsonline.org/oneworld/country_code_list.htm>

**Appendix Table S2.** Measurement of covariates.

| Variable | Measurement |
| --- | --- |
| Exact age | Continuous, years  We also categorized it into a binary variable, younger individual aged 16-59 years, or older adults aged 60 years or above |
| Sex | Binary, male or female |
| Marital status | Binary, being married or living together as married, or otherwise |
| Residence | Binary, urban or rural |
| International Immigrant | Binary, yes or no |
| Highest educational level | Current highest educational level was reported by participants according to the International Standard Classification of Education (ISCED), and we divided it into three groups in which lower group includes ISCED 0-2, middle group includes ISCED 0-2 and higher group includes ISCED 0-2. |
| Employment status | Binary, having paid employment, or otherwise |
| Income level | Participants reported their income level according to an income scale on which 1 indicates the lowest income group and 10 the highest income group in your country. We divided it into three groups in which low group includes scale 1-3, medium group includes scale 4-7 and high group includes scale 8-10. |
| Religious value | Polytomous, religious person, not religious person, or atheist |
| Attitude to science | Binary, negative or positive |
| Interested in politics | Binary, yes or no |
| Daily social media user | Binary, yes or no |

**Appendix Table S3.** The effect of COVID-19 on public confidence in the WHO: robustness tests.

| DID estimator | AOR | 95% CI | P value |
| --- | --- | --- | --- |
| **Panel A:** |  |  |  |
| Pre-pandemic × SM | 1.00 |  |  |
| After-pandemic × SM | 0.82 | 0.74–0.90 | <0.001 |
| **Panel B:** |  |  |  |
| Pre-pandemic × SI | 1.00 |  |  |
| After-pandemic × SI | 0.55 | 0.49–0.61 | <0.001 |
| **Panel C:** |  |  |  |
| Pre-pandemic × SI | 1.00 |  |  |
| After-pandemic × SI | 0.57 | 0.51–0.64 | <0.001 |
| **Panel D:** |  |  |  |
| Pre-pandemic × SI | 1.00 |  |  |
| Local epidemic × SI | 0.94 | 0.71–1.23 | 0.645 |
| Global pandemic × SI | 0.35 | 0.24–0.50 | <0.001 |

Note: AOR, the adjusted odds ratio after controlling for covariates including exact age, sex, marital status, residence, international immigrant, education level, employment status, income level, religious value, attitude to science, interested in politics, and daily social media user. Panel A using SM instead of SI to measure the severity of COVID-19; Panel B using fractional polynomial models; Panel C using sample without participants from the United; Panel D stratifying by the stage of development of the pandemic.
